# Acute myocardial infarction associated with abacavir and tenofovir based antiretroviral drug combinations in the United States

**DOI:** 10.1186/s12981-021-00383-7

**Published:** 2021-09-06

**Authors:** Kunchok Dorjee, Manisha Desai, Tsering Choden, Sanjiv M. Baxi, Alan E. Hubbard, Arthur L. Reingold

**Affiliations:** 1 Division of Epidemiology, School of Public Health, University of California Berkeley, Berkeley, CA USA; 2 Center for Tuberculosis Research, Johns Hopkins University School of Medicine, 1550 Orleans Street, Baltimore, MD 21287 USA; 3 Quantitative Sciences Unit, Center for Biomedical Informatics Research, Department of Medicine, Stanford University, Palo Alto, CA USA; 4 Department of Biomedical Data Science, Stanford University, Palo Alto, CA USA; 5 School of Public Health, Department of Epidemiology and Biostatistics, State University of New York Downstate Medical Center, Brooklyn, NY USA; 6 Department of Epidemiology and Biostatistics, University of California San Francisco, San Francisco, CA USA; 7 Division of Biostatistics, School of Public Health, University of California Berkeley, Berkeley, CA USA

**Keywords:** Human Immunodeficiency Virus, Antiretroviral agents, Cardiovascular disease

## Abstract

**Introduction:**

Although individual antiretroviral drugs have been shown to be associated with elevated cardiovascular disease (CVD) risk, data are limited on the role of antiretroviral drug combinations. Therefore, we sought to investigate CVD risk associated with antiretroviral drug combinations.

**Methods:**

Using an administrative health-plan dataset, risk of acute myocardial infarction (AMI) associated with current exposure to antiretroviral drug combinations was assessed among persons living with HIV receiving antiretroviral therapy (ART) across the U.S. from October 2009 through December 2014. To account for confounding-by-indication and for factors simultaneously acting as causal mediators and confounders, we applied inverse probability of treatment weighted marginal structural models to longitudinal data of patients.

**Results:**

Over 114,417 person-years (n = 73,071 persons) of ART exposure, 602 cases of AMI occurred at an event rate of 5.26 (95% CI: 4.86, 5.70)/1000 person-years. Of the 14 antiretroviral drug combinations studied, persons taking abacavir-lamivudine-darunavir had the highest incidence rate (IR: 11/1000; 95% CI: 7.4–16.0) of AMI. Risk (HR; 95% CI) of AMI was elevated for current exposure to abacavir-lamivudine-darunavir (1.91; 1.27–2.88), abacavir-lamivudine-atazanavir (1.58; 1.08–2.31), and tenofovir-emtricitabine-raltegravir (1.35; 1.07–1.71). Tenofovir-emtricitabine-efavirenz was associated with reduced risk (0.65; 0.54–0.78). Abacavir-lamivudine-darunavir was associated with increased risk of AMI beyond that expected of abacavir alone, likely attributable to darunavir co-administration. We did not find an elevated risk of AMI when abacavir-lamivudine was combined with efavirenz or raltegravir.

**Conclusion:**

The antiretroviral drug combinations abacavir-lamivudine-darunavir, abacavir-lamivudine-atazanavir and tenofovir-emtricitabine-raltegravir were found to be associated with elevated risk of AMI, while tenofovir-emtricitabine-efavirenz was associated with a lower risk. The AMI risk associated with abacavir-lamivudine-darunavir was greater than what was previously described for abacavir, which could suggest an added risk from darunavir. The results should be confirmed in additional studies.

**Supplementary Information:**

The online version contains supplementary material available at 10.1186/s12981-021-00383-7.

## Introduction

Over the last decade, there has been an extraordinary increase in the number of people living with HIV (PLHIV) receiving antiretroviral therapy (ART), with that number increasing from 8 million in 2010 to 25.4 million by 2019 [1]. Combination ART has been pivotal in reducing HIV/AIDS-related mortality ratios from 5.9% (1.7 million HIV/AIDS related deaths out of 28.7 million PLHIV) in 2004 to 1.8% (690,000 HIV/AIDS related deaths out of 38 million PLHIV) in 2019 [1]. As ART options expand and costs of treatment decline, understanding the toxicities associated with the use of antiretroviral agents is needed to improve health outcomes of PLHIV.

There is ongoing debate on the association of several contemporary antiretroviral agents with increased risk of cardiovascular disease, including abacavir [2–8], lamivudine [8, 9], atazanavir [9], and darunavir [10, 11]. While most antiretroviral agents are thought to contribute to cardiovascular disease by worsening blood lipid levels, other biological mechanisms, such as platelet reactivity, insulin resistance and lipodystrophy, have been reported as the underlying mechanisms for cardiotoxicity [12]. Additionally, a channeling bias may affect the observed associations between antiretroviral exposure and cardiovascular outcomes [5, 9, 13–15]. Antiretroviral medications are universally prescribed in combinations, but to date few studies have systematically evaluated the risk of cardiovascular disease associated with exposure to antiretroviral drug combinations; most CVD risk assessments have been made for individual drugs [9].

In this study, we investigated the relationship between receipts of various antiretroviral drug combinations containing agents from four major drug classes, including nucleos(t)ide reverse transcriptase inhibitors (NRTI), non-nucleoside reverse transcriptase inhibitors (NNRTI), protease inhibitors (PI), and integrase strand transfer inhibitors (InSTI), used in the treatment of HIV infection and the subsequent risk of acute myocardial infarction (AMI).

## Methods

### Study population and data

**Fig. 1:**
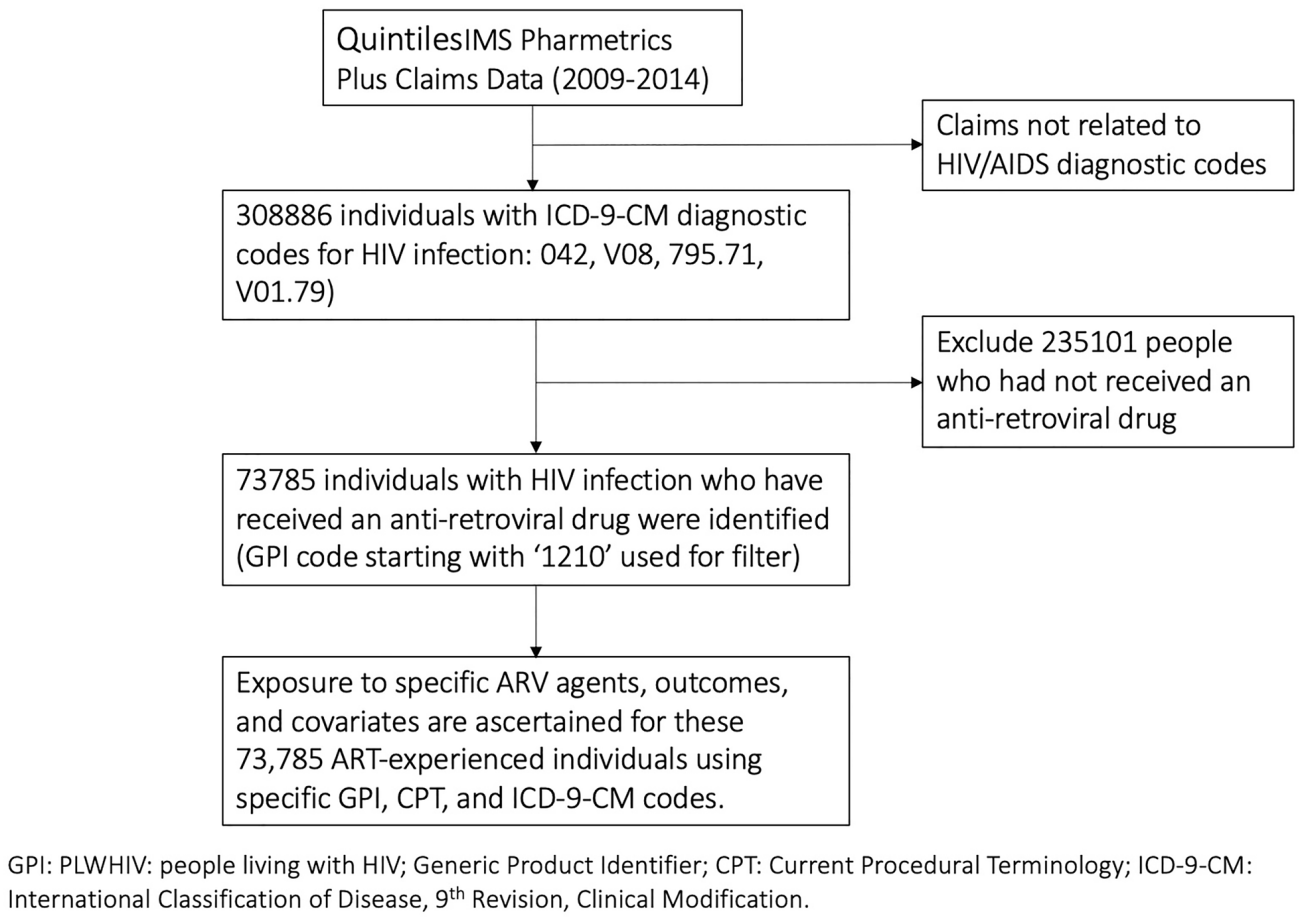
Algorithm for identification of study subjects in the Quintiles IMS Pharmetrics Plus claims data for study of cardiovascular disease associated with antiretroviral therapy

Risk of AMI associated with exposure to combinations of antiretroviral drugs was assessed among PLHIV ≥ 18 years of age started on ART between October 1, 2009 and December 31, 2014 and enrolled in the IMS’ PharMetrics Plus database in the United States. QuintilesIMS PharMetrics Plus is one of the largest health plan insurance claims databases in the U.S., containing adjudicated claims for more than 100 million unique enrollees from four regions of the U.S. [16]. In the U.S., during the study period, 98% of PLHIV were insured and thus represented by this claims dataset [16, 17]. An algorithm specified a priori was used to extract and define the study population (Fig. 1). Individuals who had not received any ART were excluded from the study. October 1, 2009 was the earliest possible date for complete availability of relevant data; ART prescription history was not available prior to this date. The baseline time point was defined as the date of ART initiation in the database and individual follow-up time was censored at the first of three events after baseline: (1) first occurrence of AMI, (2) last recorded date of ART receipt, 3) December 31, 2014. The study was approved by the Committee for Protection of Human Subjects at the University of California, Berkeley.

### Exposure and outcome

Individual antiretroviral drugs in the database were identified by their unique generic product identifier (gpi) codes. Any two prescriptions for a given antiretroviral agent separated by < 30 days were combined to represent a single continuous exposure; gaps ≥ 30 days were treated as separate ART exposures and not considered as a single continuous exposure. Start and stop dates are available for each antiretroviral drug; this enables determination of whether a specific antiretroviral drug(s) was switched to another drug. Exposure time for a drug combination was generated based on whether an individual was receiving the component drugs simultaneously, determined by examining the start and stop dates of the individual drugs. Of 23 drug combinations defined a priori, 14 combinations were assessed based on availability of adequate exposure-time (~ 1500 person-years), ascertained by examining the duration of exposure given by the dates of initiation and stopping of the antiretroviral agent in the database. Each drug combination consisted of three (or four if boosted) drugs: two NRTIs and either a boosted PI, an NNRTI, an InSTI or another NRTI. A separate analytical dataset was generated for each exposure. The data were longitudinal and each subject’s follow-up time was divided into consecutive one-month periods during which treatment status was allowed to vary. Current exposure to a drug combination was defined as exposure (yes/no) during each one-month observation period. The exposure time of a specific drug combination was compared to exposure-time of all other antiretroviral agents in assessing the outcome. The outcome was defined as the first occurrence of acute myocardial infarction after baseline, represented in the database by International Classification of Disease, 9th Revision, Clinical Modification (ICD-9-CM) code 410. xx. (Additional file 1: Table S1).

### Statistical analysis

The risk of AMI associated with current exposure to a given drug combination was assessed using stabilized inverse probability of treatment weighted (sIPTW) marginal structural models [18]. To construct sIPTWs, four pooled logistic regression models were used, two each for the denominator and numerator as previously described [5, 15]. For the denominator, the probability of exposure initiation was first modelled as a function of baseline and time-dependent covariates. Pooled logistic regression model was fit to data up to the individual’s first month of receiving the exposure or the end of follow-up for those never exposed. The probability of continuing exposure was then modelled by fitting the model to data after the first month of exposure initiation. The treatment continuation model differed from the treatment initiation model in additionally containing a variable for past month’s exposure status. The probabilities for the numerator of the sIPTW were modelled as a function of baseline covariates only. The baseline or time-fixed covariates were sex, tobacco use (ever), substance use (ever), alcohol abuse (ever), chronic hepatitis B and C virus infections, history of stroke, history of cancer, and prior myocardial infarction. The time-dependent covariates were age, treatment year, body mass index (BMI), receipt of anti-hyperglycemic agents (sulfonylureas, biguanides, insulin, thiazolidinedione), receipt of cardiovascular medications (aspirin, beta-blocker, angiotensin converting enzyme inhibitor, angiotensin receptor blocker, calcium channel blocker, and statins), and diagnosis of chronic kidney disease (CKD), dyslipidemia, heart failure, cardiac dysrhythmia, atherosclerosis, diabetes mellitus, or hypertension. Cumulative exposure to each of abacavir, lamivudine, tenofovir, emtricitabine, zidovudine, atazanavir, and darunavir was additionally incorporated in the treatment models to generate weights excluding drugs contained in the drug-combination under investigation. Follow-up time was modelled as a function of natural cubic splines with three internal knots at 25th, 50th, and 75th percentiles.

The final marginal structural hazard model was adjusted for the sIPTW and baseline values of the following covariates: baseline age, sex, year of treatment start in the database, smoking, use of alcohol, and substance, prior heart disease or receipt of medications for heart disease, lipodystrophy, hypertension, chronic kidney disease, diabetes mellitus, and dyslipidemia. Standard errors for the treatment and marginal models were calculated robustly to account for repeated measures within an individual. We performed sensitivity analyses to determine variables to include into the treatment model and assess if the models yield qualitatively different results, as described by Cole and Hernan [19].

In secondary analyses, conventional Cox proportional hazard models were run for each exposure, adjusting for baseline and time-dependent variables that included: age, treatment year, tobacco use (ever), substance use (ever) and the following conditions at baseline: stroke, hepatitis B or C virus infections, any cancer, prior heart disease or receipt of cardiovascular medications, lipodystrophy, categorical BMI status, chronic kidney disease, diabetes mellitus, and dyslipidemia. We did not adjust for multiple comparisons, as we had a priori hypotheses based on findings in the published literature and a separate dataset was meticulously generated to test each exposure; hence, such adjustments would be unnecessarily conservative. The data were extracted from the claims databases using TERADATA (Dayton, OH), and SAS version 9.1 (SAS Institute, Cary, NC). The marginal structural models were implemented in STATA version 13.1 (StataCorp, College Station, TX) following methods described by Fewell et al. [20]. We have provided additional details of the marginal structural Cox proportional hazard models in a prior publication [5].

## Results

### Population and exposure

The database contained 114,417 person-years of exposure-time to ART contributed by 73,071 study participants. AMI occurred in 602 individuals at an event rate of 5.26 (95% CI: 4.86, 5.70) per 1000 person-years. The median age of the study population was 45 years and 81.5% were male. The median follow-up time ranged from 2.67 years for individuals receiving tenofovir-emtricitabine-darunavir to 3.58 years for those tenofovir-lamivudine-zidovudine/efavirenz. The demographic and clinical characteristics of the participants overall and stratified by AMI outcome are shown in Table 1. Traditional cardiovascular disease risk factors, i.e., higher age, male sex, hypertension, diabetes mellitus, dyslipidemia, smoking, substance abuse, pre-existing heart disease, and chronic kidney disease were more prevalent among individuals who developed AMI. Fourteen antiretroviral drug combinations, each representing ≥ 1500 person-years of exposure-time, were studied (Table 2). Incidence rates [IR (95% CI)] of AMI ranged from 3.4/1000 (3/1000–4/1000) people for tenofovir-emtricitabine-efavirenz to 11.0/1000 (7.4/1000–16/1000) people for abacavir-lamivudine-darunavir.

**Table 1:** Baseline characteristics of adults with HIV infection, with and without acute myocardial infarction (AMI) receiving antiretroviral therapy in the Pharmetrics Plus data from October 2009 through December 2014 in the United States

Characteristics	All patients (N = 73,071), n (%)	Patients without AMI (N = 72,469), n (%)	Patients with AMI (N = 602), n (%)
Age, median (IQR)	45 (38–52)	45 (38–52)	53 (48–59)
Male gender	59,514 (81.4)	58,981 (81.4)	533 (88.5)
Region			
East	17,523 (24.0)	17,354 (24.0)	169 (28.1)
Mid-West	13,532 (18.5)	13,411 (18.5)	121 (20.1)
South	33,271 (45.5)	33,014 (45.6)	257 (42.7)
West	8745 (12.0)	8690 (12.0)	55 (9.1)
Year of ART initiation			
2009	24,435 (33.4)	24,127 (33.3)	308 (51.2)
2010	9729 (13.3)	9636 (13.3)	93 (15.5)
2011	10,274 (14.1)	10,190 (14.1)	84 (14.0)
2012	8605 (11.8)	8559 (11.8)	46 (7.6)
2013	7830 (10.7)	7794 (10.8)	36 (6.0)
2014	12,198 (16.7)	12,163 (16.8)	35 (5.8)
Ever substance abuse	13,395 (18.3)	13,152 (18.2)	243 (40.4)
Ever alcohol abuse	3093 (4.2)	3039 (4.2)	54 (9.0)
Ever tobacco use	11,849 (16.2)	11,587 (16.0)	262 (43.5)
Body mass index > 24.9	1511 (2.07)	1503 (2.07)	8 (1.33)
Essential hypertension	7450 (10.2)	7336 (10.1)	114 (19.0)
Diabetes mellitus or receipt of anti-hyperglycemic agents^a^	4128 (5.7)	4052 (5.6)	76 (12.6)
Chronic Kidney Disease	729 (1.0)	701 (1.0)	28 (4.7)
Dyslipidemia	8616 (11.8)	8496 (11.7)	120 (19.9)
Lipodystrophy	224 (0.3)	220 (0.3)	4 (0.7)
Pre-existing heart disease^b^	1754 (2.4)	1688 (2.3)	66 (11.0)
Receipt of medications for heart disease^c^	14,336 (19.6)	14,074 (19.4)	262 (43.5)
History of stroke	211 (0.3)	204 (0.3)	7 (1.2)
Hepatitis B	959 (1.3)	953 (1.3)	6 (1.0)
Hepatitis C	1520 (2.1)	1500 (2.1)	20 (3.3)
History of cancer	5499 (7.5)	5442 (7.5)	57 (9.5)

^a^Sulfonylureas, biguanides, insulin, thiazolidinedione

^b^Prior myocardial infarction, heart failure, cardiac arrhythmia, and atherosclerosis

^c^Aspirin, beta-blockers, calcium channel blockers, statins, angiotensin converting enzyme inhibitors, angiotensin receptor blockers

**Table 2:** Median years of exposure and number of acute myocardial infarction (AMI) events occurring over total person-time of exposure to combinations of anti-retroviral agents

Antiretroviral drug	Median no. of years of exposure (IQR)	No. of AMI events	Person years of exposure	Incidence rate per 1000 people (95% CI)
ABC-3TC-ATV	2.75 (1.58, 4.17)	30	3951	7.6 (5.3–8.7)
ABC-3TC-DRV	2.83 (1.58, 4.08)	26	2395	11.0 (7.4–16)
ABC-3TC-ZDV	2.92 (1.75, 4.33)	19	2882	6.6 (4.2–10.3)
ABC-3TC-EFV	2.92 (1.75, 4.25)	9	2541	3.5 (1.8–6.8)
ABC-3TC-RAL	3.08 (1.75, 4.12)	20	3055	6.5 (4.2–10.1)
TDF-3TC-ZDV	3.58 (2.25, 4.67)	32	5549	5.8 (4.1–8.2)
TDF-3TC-EFV	3.58 (2.33, 4.58)	18	4529	4.1 (2.5–6.3)
TDF-3TC-DRV	3.16 (2.00, 4.33)	19	2336	8.1 (5.2–12.8)
TDF-3TC-ATV	3.25 (1.92, 4.42)	23	2843	8.1 (5.4–12.2)
TDF-FTC-ATV	2·83 (1.67, 4.17)	63	13,620	4.6 (3.6–5.9)
TDF-FTC-DRV	2·67 (1.50, 4.83)	62	10,981	5.6 (4.4–7.2)
TDF-FTC-EFV	2·92 (1.75, 4.33)	170	49,493	3.4 (3.0–4.0)
TDF-FTC-FPV	3·17 (1.83, 4.42)	13	2465	5.3 (3.1–9.1)
TDF-FTC-RAL	2·83 (1.67, 4.17)	92	13,729	6.7 (5.5–8.2)

*ABC* abacavir, *TDF* tenofovir dixoproxil fumarate, *FTC* emtricitabine, *3TC* lamivudine, *ATV* atazanavir, *DRV* darunavir, *FPV* fosamprenavir, *EFV* efavirenz, *RAL* raltegravir

### Risk of AMI

**Fig. 2:**
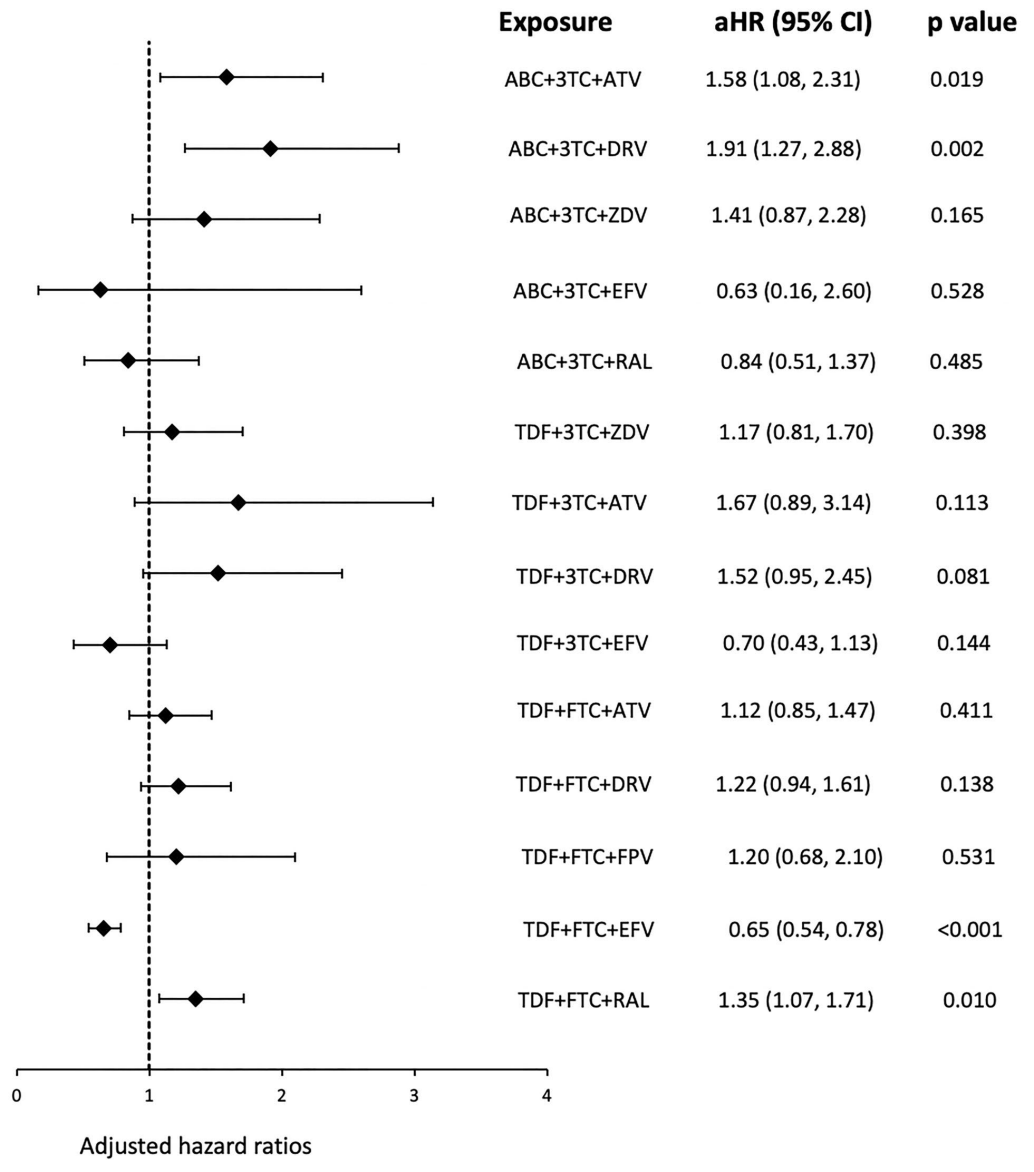
Association of various antiretroviral drug combinations with acute myocardial infarction in people living with HIV in the United States (2009–2014)

**Fig. 3:**
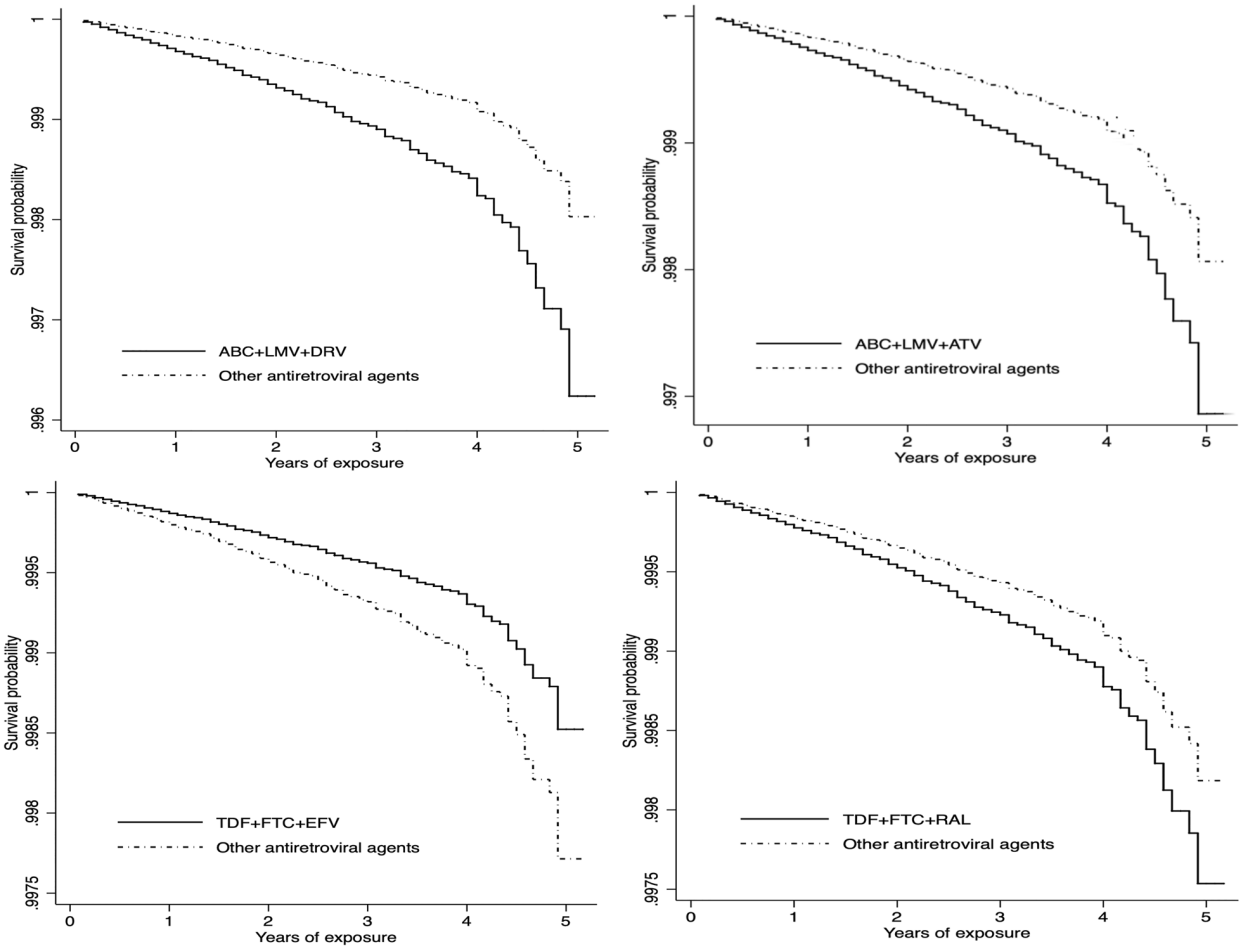
Risk of acute myocardial infarction associated with use of specific antiretroviral drug combinations in the United States (2009–2014)

Using sIPTW marginal structural models, we found significantly elevated risk of AMI for current exposure to abacavir-lamivudine-darunavir (adjusted hazard ratio: 1.91; 95% CI: 1.27–2.88, p = 0.002), abacavir-lamivudine-atazanavir (aHR: 1.58; 95% CI: 1.08- 2.31, p = 0.019), and tenofovir-emtricitabine-raltegravir (1.35; 1.07–1.71, p = 0.010). Forest plot and Kaplan Meier Survival curves for the risk of acute myocardial infarction associated with the antiretroviral drug combinations are shown in Figs. 2 and 3. Risk was not significantly elevated for the remaining regimens, including: abacavir-lamivudine-efavirenz, abacavir-lamivudine-raltegravir, abacavir-lamivudine-zidovudine, tenofovir-lamivudine-zidovudine, tenofovir-lamivudine-efavirenz, tenofovir-lamivudine-efavirenz, tenofovir-lamivudine-darunavir, tenofovir-lamivudine-atazanavir, and tenofovir-emtricitabine-fosamprenavir. Risk of AMI was lower for people receiving tenofovir-emtricitabine-efavirenz (HR: 0.65; 95% CI: 0.54–0.78, p < 0.001). Similar results were obtained in secondary analyses using conventional Cox models (Additional file 1: Table S2).

## Discussion

Using a large nationally representative real-world health plan dataset containing longitudinal information of more than 70,000 PLHIV receiving care across the United States, we have described the results of AMI risk for various antiretroviral drug combinations, several of which were recommended initial regimens. We found an elevated risk of AMI associated with current exposure to three antiretroviral drug combinations: abacavir-lamivudine-darunavir, abacavir-lamivudine-atazanavir, and tenofovir-emtricitabine-raltegravir. The incidence rate and hazard ratio were highest for abacavir-lamivudine-darunavir (IR: 11/1000 person-years; HR: 1.91) and lowest for tenofovir-emtricitabine-efavirenz (IR: 3.40/1000 person-years; HR: 0.65). The population level estimate for the annual incidence of myocardial infarction in the U.S. is 1.86/1000 people [21], whereas the AMI incidence rate for individuals receiving abacavir-lamivudine-darunavir approached the incidence rate for people ≥ 75 years of age in the U.S. (8.53–15.90/1000 person-years) [21]. In a prospective multi-national cohort (The Data Collection on Adverse Events of Anti-HIV Drugs [D:A:D]), the investigators calculated an incidence rate of 13.67/1000 person-years for PLHIV who received darunavir [11]. The association between Abacavir use and cardiovascular disease was first reported by the D:A:D study group in 2008 [8].

### Abacavir-lamivudine based combinations

There is an accumulating body of evidence suggesting an elevated risk of CVD associated with use of abacavir [3–8, 15]. PLHIV taking abacavir have previously been shown to have a 43% increase risk, using this same database [5]. The finding of a 58% increase in risk of AMI associated with the antiretroviral drug combination abacavir-lamivudine-atazanavir is in keeping with the 61% increased risk described for abacavir exposure in a recent systematic review and meta-analysis [6]. Our analysis suggests a higher risk when abacavir and lamivudine were combined with the protease inhibitor darunavir (HR: 1.91), which may suggest an incremental additive effect of darunavir. Although an analysis of data from Janssen-sponsored clinical trials suggested no elevation in risk of AMI with darunavir use [10], a CVD risk elevation of 60% for exposure to darunavir was observed in the D:A:D cohort [11]. Our finding of elevated risk for abacavir-lamivudine-atazanavir agreed with that of Desai et al. in a U.S. Veterans Affairs (VA) population [9]. The finding of no increased risk for abacavir-lamivudine-efavirenz was also observed in a prior study using a separate commercial health plan data-set in the United States that reported a significantly lower CVD risk for those taking an efavirenz-containing antiretroviral regimen, an elevated risk for efavirenz free regimens, and no risk when efavirenz was combined with abacavir [11, 22]. While it is unclear why abacavir-lamivudine-efavirenz was not associated with an elevated risk in this study, given that abacavir has been shown to be associated with CVD elsewhere; [6] interaction between abacavir and efavirenz may be possible although we are not aware of a biological or pharmacological rationale for such an interaction. We did not find an elevated risk of AMI for the abacavir-lamivudine-raltegravir combination. While it is possible that patients with unfavorable cardiovascular risk profiles may be channeled away from receiving an abacavir based regimen including abacavir-lamivudine-raltegravir, it remains to be determined if any portion of this finding may be attributable to the lipid lowering property of InSTIs [23–25]. Our investigation of the 14 drug combinations, nine of which contained lamivudine, did not reveal a pattern indicating that lamivudine in and of itself may be associated with an elevated risk of AMI. However, the D:A:D study authors reported an increased risk of AMI associated with recent exposure to lamivudine after adjusting for past exposure, and the VA study found an elevated risk of CVD for lamivudine use, noting that all of the drug-combinations associated with an elevated CVD risk contained lamivudine [8, 9]. The differing findings on lamivudine call for further investigations.

### Tenofovir-lamivudine and tenofovir-emtricitabine based combinations

We did not find any significantly increased risk of AMI for most of the tenofovir-containing combinations, including tenofovir-lamivudine-efavirenz, tenofovir-emtricitabine-atazanavir, and tenofovir-emtricitabine-fosamprenavir, tenofovir-lamivudine-atazanavir, tenofovir-lamivudine-darunavir, and tenofovir-emtricitabine-darunavir. Our findings on tenofovir-based regimens largely agree with that of the VA study. We are not aware of a prior study that has implicated tenofovir/emtricitabine in the development of AMI. Tenofovir has been shown to have an intrinsic lipid lowering effect and to decrease the carotid intima media thickness, suggesting possible cardiovascular and cerebrovascular protective effects [26, 27]. We observe an increased risk of AMI (HR:1.35; 95% CI: 1.07–1.71)) when tenofovir-emtricitabine was combined with raltegravir. This finding prompted us to assess the distribution of baseline CVD risk factors for the participants who did and did not receive tenofovir-emtricitabine-raltegravir. We observed that participants who received tenofovir-emtricitabine-raltegravir during the follow-up period were more likely to have cardiovascular disease and dyslipidemia at baseline, compared to participants who did not receive this combination (14 vs 11%, p value < 0.001). Therefore, it is possible that individuals with an adverse cardiovascular disease risk profile may have selectively been given tenofovir-emtricitabine-raltegravir, in an effort to reduce their ART-attributable CVD risk. Also, we note the possibility of advanced HIV disease, which is independently associated with CVD risk [28], in patients receiving tenofovir-emtricitabine-raltegravir. Our dataset lacks CD4 cell-count and HIV viral load data and therefore, we could not investigate this hypothesis. Nevertheless, we are not aware of a prior study that has investigated the risk of CVD among recipients of an antiretroviral drug that contains raltegravir, and encourage further inquiry to determine if this observation and that of a risk reduction with EFV, are due to random chance or channeling bias.

Our findings on the drug combinations have implications for clinical practice, as they inform the thinking about risk of CVD in the setting of combination ART—the only way in which ART is currently used. The combination of abacavir-lamivudine and dolutegravir, an InSTI, is a recommended initial ART regimen for PLHIV. Our finding of no increased risk when abacavir-lamivudine was combined with raltegravir suggests value in further study of other InSTI-based combinations including abacavir-lamivudine-dolutegravir. However, we note that raltegravir and dolutegravir, although both being InSTIs, can be quite different in terms of their side-effect profiles [29]. We encourage further investigations and external validation of raltegravir and its combinations with abacavir-lamivudine or tenofovir-emtricitabine, especially as both combinations with InSTIs are recommended as initial ART regimens for PLHIV [30].

The claims database we used has limitations that should be noted. First, there is no information on possible risk modifiers, such as CD4 cell-count, HIV viral load and race/ethnicity. We are unsure how these factors may have influenced the prescription of a tenofovir or abacavir based antiretroviral regimen. Also, the majority of the study population are male and therefore, the results may not be readily generalizable to women living with HIV. Although, the ICD-9-CM code used to ascertain AMI outcome has been previously validated, [31] some misclassification of events may have occurred. We are unsure whether any such misclassification would have affected the direction of the associations of various antiretroviral drug combinations with CVD risk in the participants. ART prescription history prior to October 1, 2009 was also not available in the database. As a result, influence of prior exposure to the older protease inhibitors and to thymidine analogues on the subsequent development of CVD could not be assessed. Data were generated from claims submitted to the health plan for reimbursement, and it is possible that information on health conditions for which reimbursement may not be sought could be under-reported in the database. However, such errors are likely to affect exposure groups non-differentially, in which case any bias in the results would have been toward the null. Coding errors and missing data related to treatment adherence are most likely to have affect the exposure groups non-differentially. Finally, in the modeling, we could not have been able to adjust for unmeasured confounders or residual confounding, even with marginal structure models, which are entirely dependent on the quality of data at entry.

There are a number of strengths to this work. Most studies on CVD risk from ART exposure so far have been in clinical cohorts; validating risk in a different setting is important. Compared to prior studies, we used more recent data. Because of the possibility of confounding-by-indication where an individual receives (or not) a drug based on specific clinical indications, such as chronic kidney disease or dyslipidemia, we used inverse-probability weighted marginal structural models to account for confounding. We have reported our results from marginal structural and conventional Cox models. Although the estimates from marginal structural models and Cox models did not differ substantially, the former seeks to capture the CVD risk had all PLHIV received the given antiretroviral drug combination vs had they all not received the ART combination. Hence, the estimates from the marginal models may be more representative of the true population estimates [18, 32].

## Conclusion

The antiretroviral drug combinations of abacavir-lamivudine-darunavir, abacavir-lamivudine-atazanavir, and tenofovir-emtricitabine-raltegravir were found to be associated with a greater risk of AMI, whereas tenofovir-emtricitabine-efavirenz was found to be associated with a lower risk of AMI. The AMI risk for abacavir-lamivudine-darunavir was greater than what has been previously described for abacavir, suggesting an added risk from darunavir. The results should be confirmed in additional studies. Greater awareness of the effects of specific antiretroviral drug combinations can benefit patients with specific risk profiles, ART history, or antiretroviral drug contraindications.

## Supplementary Information

**Additional file 1**: **Table S1**. ICD-9-CM, and CPT codes for defining various covariates and outcomes. **Table S2**. Risk of myocardial infarction associated with current exposure to various combinations of antiretroviral agents among people living with HIV in the United States

## Data Availability

No restrictions.
