# Supplementary material for: Acute myocardial infarction associated with abacavir and tenofovir based antiretroviral drug combinations in the United States
Source: AIDS Res Ther. 2021 Sep 6;18:57. doi: 10.1186/s12981-021-00383-7 (PMC8419948; doi:10.1186/s12981-021-00383-7)
Supplement: Supplementary file 1 — Additional file 1: Table S1. ICD-9-CM, and CPT codes for defining various covariates and outcomes. Table S2. Risk of myocardial infarction associated with current exposure to various combinations of antiretroviral agents among people living with HIV in the United States [file 12981_2021_383_MOESM1_ESM.docx]

**Supplemental Web Material**

**Contents:**

**Supplemental Table 1. (Page 1)**

ICD-9-CM, and CPT codes for defining various covariates and outcomes

**Supplemental Table 2. (Page 2)**

Risk of myocardial infarction associated with current exposure to various combinations of antiretroviral agents among people living with HIV in the United States

**Supplemental Table 1**.

| **ICD-9-CM codes used for defining various covariates and outcome** | |
| --- | --- |
| **Variable** | **ICD-9-CM Code** |
| Acute myocardial infarction | 410.xx |
| Tobacco use | 305.1; v15.82 |
| Substance abuse (dependent and non-dependent) | 304.xx, 305.xx |
| Alcohol abuse (alcohol dependence and alcohol abuse) | 303.xx, 305.0x |
| Overweight/obese | 278.00, 278.01, 278.02 |
| Diabetes mellitus | 250.xx, 357.2x, 362.0x, 366.41 |
| Essential hypertension | 401.xx |
| Hypercholesterolemia | 272.0x |
| Hypertriglyceridemia | 272.1x |
| Mixed hyperlipidemia | 272.2x |
| Other and unspecified hyperlipidemia | 272.4x |
| Lipodystrophy | 272.6x |
| Chronic kidney disease | 585.xx |
| Heart failure | 402.01, 402.91, 428.0, 404.01, 404.03,404.11,404.13,404.91, 404.93 |
| Cardiac dysrhythmia | 427.xx |
| Old myocardial infarction | 427.xx |
| Coronary atherosclerosis | 414.xx |
| Stroke | 434.xx |
| Hepatitis B virus infection | 070.2x, 070.3x, V02.61 |
| Hepatitis C virus infection | 070.41, 070.44, 070.51, 070.54, 070.7x, v02.62 |
| Any cancer | 140-149, 150-159, 160-169, 170-179, 180-189, 190-199, 200-209, 210-229, 230-239 |

ICD-9-CM: International Classification of Disease, 9th Revision, Clinical Modification.

**Supplemental Table 2.**

| **Risk of myocardial infarction from current exposure to combinations of antiretroviral agents as compared to others among HIV-infected individuals receiving ART** | | | |
| --- | --- | --- | --- |
| **Anti-retroviral**  **drug** | **Unadjusted Cox Model**  **HR (95% CI; p value)** | **Adjusted Cox Model**  **HR^#^ (95% CI; p value)** | **Marginal Structural Model^*^**  **HR (95% CI; p value)** |
| ABC+3TC+ATV | 1.63 (1.13, 2.36; p=0·009) | 1·44 (0.99, 2.08; p=0·056) | 1.58 (1.08, 2.31; p=0.019) |
| ABC+3TC+DRV | 2.34 (1.58, 3.47; p<0·001) | 1·87 (1.25, 2.79; p=0·002) | 1.91 (1.27, 2.88; p=0.002) |
| ABC+3TC+ZDV | 1.41 (0.89, 2·.3; p=0.140) | 1·30 (0·80, 2.01; p=0·306) | 1.41 (0.87, 2.28; p=0.165) |
| ABC+3TC+EFV | 0·74 (0.38, 1.43; p=0.375) | 0·57 (0·30, 1.11; p=0·099) | 0.63 (0.16, 2.60; p=0.528) |
| ABC+3TC+RAL | 1.40 (0.90, 2.19; p=0.136) | 1.01 (0.64, 1.59; p=0.966) | 0.84 (0.51, 1.37; p=0.485) |
| TDF+3TC+ZDV | 1.23 (0.86, 1.76; p=0.249) | 1.23 (0.86, 1.77; p=0.252) | 1.17 (0.81, 1.70; p=0.398) |
| TDF+3TC+ATV | 1.74 (1.15, 2.65; p=0.009) | 1.61 (1.06, 2.45; p=0.027) | 1.67 (0.89, 3.14; p=0.113) |
| TDF+3TC+DRV | 1.75 (1.11, 2.76; p=0.017) | 1.54 (0.97, 2.45; p=0.065) | 1.52 (0.95, 2.45; p=0.081) |
| TDF+3TC+EFV | 0.84 (0.53, 1.34; p=0.468) | 0.76 (0.47, 1.22; p=0.256) | 0.70 (0.43, 1.13; p=0.144) |
| TDF+FTC+ATV | 0.97 (0.73, 1.25; p=0.712) | 1.05 (0.81, 1.37; p=0.717) | 1·12 (0·85, 1·47; p=0·411) |
| TDF+FTC+DRV | 1.20 (0.92, 1.56; p=0.178) | 1.20 (0.92, 1.57; p=0.171) | 1·22 (0·94, 1.61; p=0·138) |
| TDF+FTC+FPV | 1.11 (0.64, 1.93; p=0·710) | 1.13 (0.65, 1.96; p=0.659) | 1·20 (0·68, 2.10; p=0·531) |
| TDF+FTC+EFV | 0.60 (0.50, 0.72; p<0.001) | 0.61 (0.51, 0.74; p<0.001) | 0.65 (0.54, 0.78; p<0.001) |
| TDF+FTC+RAL | 1.47 (1.18, 1.84; p=0.001) | 1.37 (1.10, 1.72; p=0.006) | 1.35 (1.07, 1.71; p=0.010) |

^#^Adjusted for baseline/time-fixed covariates gender, tobacco use (ever), substances or alcohol abuse (ever), serologic evidence of hepatitis B & C infections, history of stroke, cancer, prior myocardial infarction, heart disease (atherosclerosis, congestive heart failure, cardiac dysrhythmias), chronic kidney disease, hypertension, lipodystrophy, dyslipidemia, and diabetes mellitus, cardiovascular medications (aspirin, beta-blocker, angiotensin converting enzyme inhibitor, angiotensin receptor blocker, calcium channel blocker, and statins), anti-hyperglycemic medications (sulfonylureas, biguanides, insulin, thiazolidinedione), and time-dependent covariates age and treatment year.

^*^Adjusted for weights generated from treatment models as a function of time-fixed covariates listed above and time-dependent covariates age, treatment year, heart disease, chronic kidney disease, hypertension, lipodystrophy, dyslipidemia, diabetes mellitus, cardiovascular medications, and anti-hyperglycemic medications. In addition to the stabilized treatment weight, the marginal structural model was additionally adjusted for gender and baseline values of the time-updated covariates age, heart disease, chronic kidney disease, hypertension, lipodystrophy, dyslipidemia, and diabetes mellitus, cardiovascular medications anti-hyperglycemic medications.

ABC: abacavir; 3TC: lamivudine; ATV: atazanavir; DRV: darunavir; FPV: fosamprenavir; TDF: tenofovir; FTC: emtricitabine; EFV: efavirenz; RAL: raltegravir;
